# A conceptual model for optimizing vaccine coverage to reduce vector-borne infections in the presence of antibody-dependent enhancement

**DOI:** 10.1186/s12976-018-0085-x

**Published:** 2018-09-03

**Authors:** Biao Tang, Xi Huo, Yanni Xiao, Shigui Ruan, Jianhong Wu

**Affiliations:** 1 School of Mathematics and Statistics, Xi’an Jiaotong University, Xi’an, 710049 People’s Republic of China; 2 Centre for Disease Modelling, Laboratory for Industrial and Applied Mathematics, York University, Toronto, M3J 1P3 Canada; 3 Department of Mathematics, University of Miami, Coral Gables, 33146 USA

**Keywords:** Zika, Dengue, Antibody dependent enhancement, Optimized vaccination strategies, Mathematical modelling

## Abstract

**Background:**

Many vector-borne diseases co-circulate, as the viruses from the same family are also transmitted by the same vector species. For example, Zika and dengue viruses belong to the same *Flavivirus* family and are primarily transmitted by a common mosquito species *Aedes aegypti*. Zika outbreaks have also commonly occurred in dengue-endemic areas, and co-circulation and co-infection of both viruses have been reported. As recent immunological cross-reactivity studies have confirmed that convalescent plasma following dengue infection can enhance Zika infection, and as global efforts of developing dengue and Zika vaccines are intensified, it is important to examine whether and how vaccination against one disease in a large population may affect infection dynamics of another disease due to antibody-dependent enhancement.

**Methods:**

Through a conceptual co-infection dynamics model parametrized by reported dengue and Zika epidemic and immunological cross-reactivity characteristics, we evaluate impact of a hypothetical dengue vaccination program on Zika infection dynamics in a single season when only one particular dengue serotype is involved.

**Results:**

We show that an appropriately designed and optimized dengue vaccination program can not only help control the dengue spread but also, counter-intuitively, reduce Zika infections. We identify optimal dengue vaccination coverages for controlling dengue and simultaneously reducing Zika infections, as well as the critical coverages exceeding which dengue vaccination will increase Zika infections.

**Conclusion:**

This study based on a conceptual model shows the promise of an integrative vector-borne disease control strategy involving optimal vaccination programs, in regions where different viruses or different serotypes of the same virus co-circulate, and convalescent plasma following infection from one virus (serotype) can enhance infection against another virus (serotype). The conceptual model provides a first step towards well-designed regional and global vector-borne disease immunization programs.

## Background

Our conceptual modelling study is motivated by the observation that several vector-borne diseases (or several serotypes of the same disease) may share the same vector species, and convalescent plasma following infection of one disease (or one serotype) can enhance the infection to another disease (or another serotype). We wish to address the following hypothetical issue: if a vaccine product for one particular disease (or a particular serotype) becomes available and if the aforementioned antibody-dependent enhancement does occur, is there an optimal vaccine coverage that can control the outbreak of the particular disease while simultaneously contributing to the control of other diseases (or serotypes) in the presence of antibody enhancement.

Our conceptual model formulation is guided by Zika outbreaks in dengue endemic areas. Dengue fever is caused by any of four closely related viruses or serotypes (DENV 1, DENV 2, DENV 3, DENV 4) and is transmitted between people by *Aedes aegypti* mosquitoes which are found throughout the world. Today about 2.5 billion people live in areas where there is a risk of dengue transmission with 50-100 million infections yearly, including 500,000 dengue hemorrhagic fever (DHF) cases and 22,000 deaths [1–3]. The antigenic differences among four serotypes are so great that robust immunity to one conferred by recovery from infection does not confer immunity to the others. Instead, previous exposure to one serotype increases the risk of severe disease after infection by a second serotype, the phenomenon of antibody-dependent enhancement (ADE) [4–6]. Studies based on modeling multiple DENV strains indicate that preexisting antibodies can significantly affect the dengue viral dynamics and disease transmission [7–9]. The cross-reactivity and ADE have been imposing substantial challenges for the development of an ideal dengue vaccine since it needs to balance protective response against all four serotypes. This is illustrated by the experience of the first dengue vaccine, Dengvaxia produced by *Sanofi Pasteur*, that was approved for use in six countries [10], and WHO published the recommendations of the Strategic Advisory Group of Experts (SAGE) on Immunization on the use of Dengvaxia in May 2016. However, following the disclosure to WHO of additional data by *Sanofi Pasteur*, WHO initiated a process engaging independent external experts [11], and this process led to revised recommendations from SAGE on April 18 of 2018.

Zika virus (ZIKV), also a member of the *Flavivirus* family, was first isolated from a rhesus monkey in the Zika forest of Uganda in 1947 [12]. The first severe ZIKV outbreak occurred on Yap Island in the North Pacific in 2007 [13]. In 2013-2014, large-scale ZIKV outbreaks were reported on other Pacific islands, including French Polynesia, New Caledonia, Easter Island, and Cook Island [14, 15]. After being transmitted to Brazil in 2015 [16], ZIKV was subsequently spread to other countries and territories in the Americas, and was estimated to become a potential threat to countries in Europe [17], Africa and the Asia-Pacific region [18, 19]. By December 29 of 2016, 48 countries and territories in the Americas had confirmed autochthonous vector-borne transmission of ZIKV disease with more than 520,000 suspected cases [20]. Though non-vector borne transmission such as sexual transmission [21] and vertical transmission [22] has been reported, ZIKV is primarily transmitted by the bite of infected *Aedes aegypti* mosquitoes, the same mosquito species that transmits dengue viruses.

Since ZIKV outbreaks usually occurred in areas where dengue was endemic, cocirculation and coinfection of dengue and Zika has been reported [23, 24], and since there is evidence that immunological cross-reactivity occurs between dengue and Zika and the ADE of dengue viruses can enhance Zika infections [25–27], it is natural to ask whether and how dengue vaccine (when available) utilization in a population impacts Zika infection dynamics [28, 29].

A previous study [30] reported that dengue vaccine may increase Zika infections. This study was based on the assumption of a very high effective vaccine coverage rate. Since the effective vaccine rate is the vaccine coverage rate times the vaccine efficacy while the vaccine efficacy of existing vaccine candidates is moderate, the effective vaccine rate is moderate in real settings. Hence, it is natural to ask if a large-scale use of DENV vaccine with moderate effective vaccine rate feasible in real settings would increase the likelihood of ZIKV outbreak and lead to a larger number of ZIKV infections in the population. Our analysis provides a negative answer to this question, so we are led to ask *if there is an optimal DENV vaccine coverage rate with which the dengue vaccination program not only controls the dengue transmission but also reduces ZIKV infections*.

The main objective of this study is to address this question through a deterministic model for the coinfection of DENV and ZIKV among mosquitos and humans. We perform intensive simulations on a wide range of the basic reproduction numbers of dengue and Zika reported from different areas in the world, and show that under a wide range of circumstances, the use of a dengue vaccine in the population can be designed to not only help control the dengue outbreak but also, counter-intuitively, reduce Zika infections. We remark that this conclusion is based on a hypothetical dengue vaccine being used in a population in a dengue epidemic area with a particular serotype.

## Methods

**Fig. 1:**
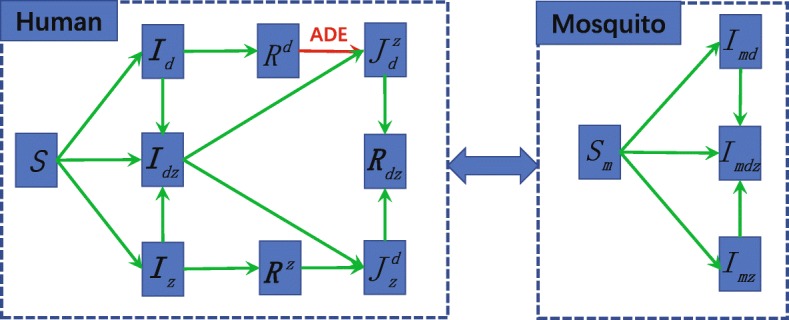
Model compartmental diagram

All our mathematical analyses and numerical simulations are based on the model described in Fig. 1 which is formulated in system (1)-(2) with parameters illustrated in Table 1. In particular, the mosquito population *N*_*m*_ is divided into compartments of susceptible, infected with dengue only, infected with Zika only, infected with both dengue and Zika, and their population densities are respectively denoted by *S*_*m*_,*I*_*md*_,*I*_*mz*_,*I*_*mdz*_.

**Table 1:** Parameter definitions and values

	Definitions	Value(range)	Reference
*c*	Mosquito biting rate	0.8(0.3,1)	[21, 35]
*β*_*d*_	Mosquito-to-human transmission probability of dengue	Varied ^∗^ (0.045,0.32)	[21, 35]
*β*_*z*_	Mosquito-to-human transmission probability of Zika	Varied ^∗^ (0.125,0.281)	[21, 35]
*η*_*d*_	Human-to-mosquito transmission probability of dengue	0.5(0.3,0.75)	[21, 35]
*η*_*z*_	Human-to-mosquito transmission probability of Zika	0.5(0.3,0.75)	[21, 35]
*γ*_*d*_	Recovery rate of humans infected with dengue	0.2(0.017,0.33)	[35]
*γ*_*z*_	Recovery rate of humans infected with Zika	0.2(0.14,0.33)	[36–38]
*μ*	Mosquito mortality rate	0.1(0.028,0.25)	[21, 35]
*κ*	Antibody dependent enhancement/neutralization factor	Varied (0,3)	[7–9, 32, 33]
	of the susceptibility of ZIKV		

^*^ *β*_*d*_ and *β*_*z*_ are calculated from varied values of *R*_*d*_∈(1.2,3.2) and *R*_*z*_∈(2,3)

In particular, the model equations for the mosquito population are given by

1 $$ {\begin{aligned} &\frac{d S_{m}}{dt}=\Lambda - c\left(\eta_{d}I_{d}+\eta_{z}I_{z}+\left(\eta_{d} + \eta_{z} - \eta_{d} \eta_{z} \right) I_{dz}+\eta_{z}J^{z}_{d}+\eta_{d}J^{d}_{z}\right)\frac{S_{m}}{N_{h}}-\mu S_{m},\\ &\frac{d I_{md}}{dt}=c \left(\eta_{d} I_{d} + \eta_{d} (1-\eta_{z}) I_{dz}+\eta_{d}J^{d}_{z}\right)\frac{S_{m}}{N_{h}}-c \eta_{z} \left(I_{z}+I_{dz}+J^{z}_{d}\right)\frac{I_{md}}{N_{h}}-\mu I_{md},\\ &\frac{d I_{mz}}{dt}=c \left(\eta_{z}I_{z}+\eta_{z} (1-\eta_{d})I_{dz}+\eta_{z}J^{z}_{d}\right)\frac{S_{m}}{N_{h}}-c \eta_{d} \left(I_{d}+I_{dz}+J^{d}_{z}\right)\frac{I_{mz}}{N_{h}}-\mu I_{mz},\\ &\frac{d I_{mdz}}{dt}=c \eta_{d} \eta_{z} I_{dz} \frac{S_{m}}{N_{h}} + c \eta_{z} \left(I_{z}+I_{dz}+J^{z}_{d}\right)\frac{I_{md}}{N_{h}}+ c \eta_{d} \left(I_{d}+I_{dz}+J^{d}_{z}\right)\frac{I_{mz}}{N_{h}} - \mu I_{mdz}, \end{aligned}}  $$

where *Λ* is the recruitment rate of mosquitoes, *μ* is the mosquito mortality rate, and *c* is the mosquito daily biting rate. {*η*_*i*_}_*i*=*d*,*z*_ is the human to mosquito transmission probability of disease *i* per contact. Specifically, during a contact between a susceptible mosquito and a co-infected human, the probability of the mosquito getting contaminated by dengue, Zika, and both viruses are respectively *η*_*d*_(1−*η*_*z*_), (1−*η*_*d*_)*η*_*z*_, and *η*_*d*_*η*_*z*_.

The human population *N*_*h*_ is divided into compartments of individuals that are susceptible (*S*), infected by dengue alone (*I*_*d*_), infected by Zika alone (*I*_*z*_), coinfected by both dengue and Zika (*I*_*dz*_), immune to dengue (*R*^*d*^), immune to Zika (*R*_*z*_), immune to dengue and infected by Zika $\left (J_{d}^{z}\right)$, immune to Zika and infected by dengue $\left (J_{z}^{d}\right)$, and immune to both diseases (*R*_*dz*_). In our model, the Zika infected classes *I*_*z*_, *I*_*dz*_, and $J_{d}^{z}$ include both symptomatic and asymptomatic individuals. The model equations for the transmission of dengue and Zika among humans take the following form:

2 $$ {\begin{aligned} &\frac{dS}{dt}=-c\left(\beta_{d}I_{md}+\beta_{z}I_{mz}+\left(\beta_{d}+\beta_{z}-\beta_{d} \beta_{z}\right)I_{mdz}\right)\frac{S}{N_{h}},\\ &\frac{dI_{d}}{dt}=c\left(\beta_{d}I_{md}+\beta_{d} (1-\beta_{z}) I_{mdz}\right)\frac{S}{N_{h}}-c \beta_{z}(I_{mz}+I_{mdz})\frac{I_{d}}{N_{h}}-\gamma_{d} I_{d},\\ &\frac{dI_{z}}{dt}=c\left(\beta_{z}I_{mz}+\beta_{z}(1-\beta_{d}) I_{mdz}\right)\frac{S}{N_{h}}-c \beta_{d}\left(I_{md}+I_{mdz}\right)\frac{I_{z}}{N_{h}}-\gamma_{z} I_{z},\\ &\frac{dI_{dz}}{dt}=c\beta_{d} \beta_{z} I_{mdz}\frac{S}{N_{h}}+c \beta_{z}(I_{mz}+I_{mdz})\frac{I_{d}}{N_{h}} + c \beta_{d}(I_{md}+I_{mdz})\frac{I_{z}}{N_{h}} - (\gamma_{d}+\gamma_{z}) I_{dz},\\ &\frac{dR^{d}}{dt}=\gamma_{d} I_{d} - \kappa c \beta_{z} (I_{mz}+I_{mdz})\frac{R^{d}}{N_{h}},\\ &\frac{dR^{z}}{dt}=\gamma_{z} I_{z} - c \beta_{d} (I_{md}+I_{mdz})\frac{R^{z}}{N_{h}},\\ &\frac{dJ_{d}^{z}}{dt}=\kappa c \beta_{z} (I_{mz}+I_{mdz})\frac{R^{d}}{N_{h}}+\gamma_{d}I_{dz}-\gamma_{z} J_{d}^{z},\\ &\frac{dJ_{z}^{d}}{dt}=c \beta_{d} (I_{md}+I_{mdz})\frac{R^{z}}{N_{h}} + \gamma_{z} I_{dz}-\gamma_{d} J_{z}^{d},\\ &\frac{dR_{dz}}{dt}=\gamma_{z} J_{d}^{z}+ \gamma_{d} J_{z}^{d}, \end{aligned}}  $$

where {*γ*_*i*_}_*i*=*d*,*z*_ is the human recovery rate from disease *i*, and {*β*_*i*_}_*i*=*d*,*z*_ is the mosquito to human transmission probability of disease *i* only per contact. Thus during a contact between a susceptible human and a mosquito with both viruses, the probability of the human getting infected by dengue, Zika, and both viruses are respectively *β*_*d*_(1−*β*_*z*_), (1−*β*_*d*_)*β*_*z*_, and *β*_*d*_*β*_*z*_.

In this study, we assume the human population *N*_*h*_ remains a constant.

### Basic reproduction numbers

Relevant to the infection dynamics characteristics is the well-known dengue and Zika basic reproduction numbers, *R*_*d*_ and *R*_*z*_, in a given region. The basic reproduction number is the average number of total infections generated by the introduction of a single infected individual into the population. These two basic reproduction numbers respectively measure the initial growth rates of dengue and Zika outbreaks. Calculations of the basic reproductive numbers can be done following the method described in [31] through multiple steps similar to those in [30]. We obtain that the basic reproduction number of system (1)–(2) is *R*_0_= max{*R*_*d*_,*R*_*z*_}, where

$$R_{d}=\sqrt{\frac{c\beta_{d}}{\mu}\frac{\Lambda c\eta_{d}}{\mu N_{h}\gamma_{d}}}\quad \text{and}\quad R_{z}=\sqrt{\frac{c\beta_{z}}{\mu}\frac{\Lambda c\eta_{z}}{\mu N_{h}\gamma_{z}}} $$

are respectively the dengue and Zika basic reproduction numbers. Thus there will be no outbreak if *R*_0_<1, and there will be outbreaks of dengue or Zika if the corresponding basic reproduction number exceeds 1.

A dengue vaccination will change the above basic reproduction numbers to the so-called control reproduction numbers, denoted by $R_{d}^{P_{v}}$ and $R_{z}^{P_{v}}$, respectively, where the upper index *P*_*v*_ represents the effective vaccine coverage rate (the vaccine coverage times the vaccine efficacy). With an effective dengue vaccine coverage rate *P*_*v*_, the control reproduction numbers are

$$R_{d}^{P_{v}}=\sqrt{\frac{c\beta_{d}(1-P_{v})}{\mu}\frac{\Lambda c\eta_{d}}{\mu N_{h}\gamma_{d}}} $$

and

$$R_{z}^{P_{v}}=\sqrt{\frac{c\beta_{z}(1-P_{v})}{\mu}\frac{\Lambda c\eta_{z}}{\mu N_{h}\gamma_{z}}+\frac{c\kappa\beta_{z}P_{v}}{\mu}\frac{\Lambda c\eta_{z}}{\mu N_{h} \gamma_{z}}}, $$

where *κ* is the ADE degree as described in the following section. Clearly, dengue vaccination reduces outbreak possibility of dengue since $R_{d}^{P_{v}}<R_{d}$, but increases the outbreak potential of Zika if *κ*>1.

### Parameter set up

As mentioned above, we use parameter *κ* to measure the ADE induced multiplication factor of the susceptibility to ZIKV. Thus in our model, the Zika infection force for people immunized to dengue is amplified by *κ* compared to the Zika infection force for people who have never had dengue infection or immunization, and *κ*>1 [7, 25–27]. We assume that dengue immunity from natural infection and vaccination are immunologically identical, that is, both dengue-recovered and effectively dengue-vaccinated individuals are immune to dengue, and have the same degree of ADE for Zika infections. To what extent this assumption holds depends on the DENV serotype and the vaccine characteristics, and we will discuss this in the final section.

ADE and antibody-dependent neutralization (ADN) also exist among the four serotypes of dengue viruses, of which the multiplication factors of susceptibility have been studied and estimated using epidemiological data [7–9, 32, 33]. Although the ADE effect of dengue on Zika infection has been observed in cellular level experiments [25, 26, 34], the actual ADE factor *κ* is difficult to be estimated from the experimental measurements [7]. So we adopt the parameter value *κ* estimated/assumed in the aforementioned dengue studies as *κ*∈[0,3], complemented by an intensive sensitivity analysis.

In our simulations, we fix the following parameter values: (1) the transmission probabilities from human to mosquito of both diseases are assumed as a constant value *η*_*d*_=*η*_*z*_=0.5; (2) The human recovery rates from both diseases are fixed as *γ*_*d*_=*γ*_*z*_=0.2day^−1^; (3) the total human population is fixed as *N*_*h*_=100,000, the recruitment rate of mosquito population is fixed as *Λ*=20,000. Table 1 gives the overview of the parameter ranges and literatures from which they are cited.

We vary the basic reproduction numbers *R*_*d*_ from 1.2 to 3.2 and *R*_*z*_ from 2 to 3 by adjusting values of *β*_*d*_ and *β*_*z*_ in credible ranges in agreements with those from [21, 35]. These basic reproduction number values are in broad agreement with previous dengue [21, 35] and Zika [21, 36–38] estimates.

When dengue vaccine is used, the initial values of the compartments *S* and *R*^*d*^ are changed accordingly while the others remain unchanged. Specifically, we use the following initial values for system (1)–(2) for the scenario with no vaccination:

$$\begin{aligned} S(0)&=N_{h}-200, I_{d}(0)=100, I_{z}(0)=100, S_{m}(0)=10^{5},\\ I_{dz}(0)&=R^{d}(0)=R^{z}(0)=J_{d}^{z}(0)=J_{z}^{d}(0)=R_{dz}(0)=0,\\ I_{md}(0)&=I_{mz}(0)=I_{mdz}(0)=0. \end{aligned} $$

For the scenario when a percentage of *P*_*v*_ human population is effectively covered by dengue vaccination at the onset of the outbreak, the initial conditions of compartments *S* and *R*^*d*^ are modified as $\hat {S}(0)=(1-P_{v})(N_{h}-200)$ and $\hat {R}^{d}(0)=P_{v} (N_{h}-200)$ while the other components remain unchanged.

## Results

### Potential impact of dengue vaccination on the final size of Zika infections

**Fig. 2:**
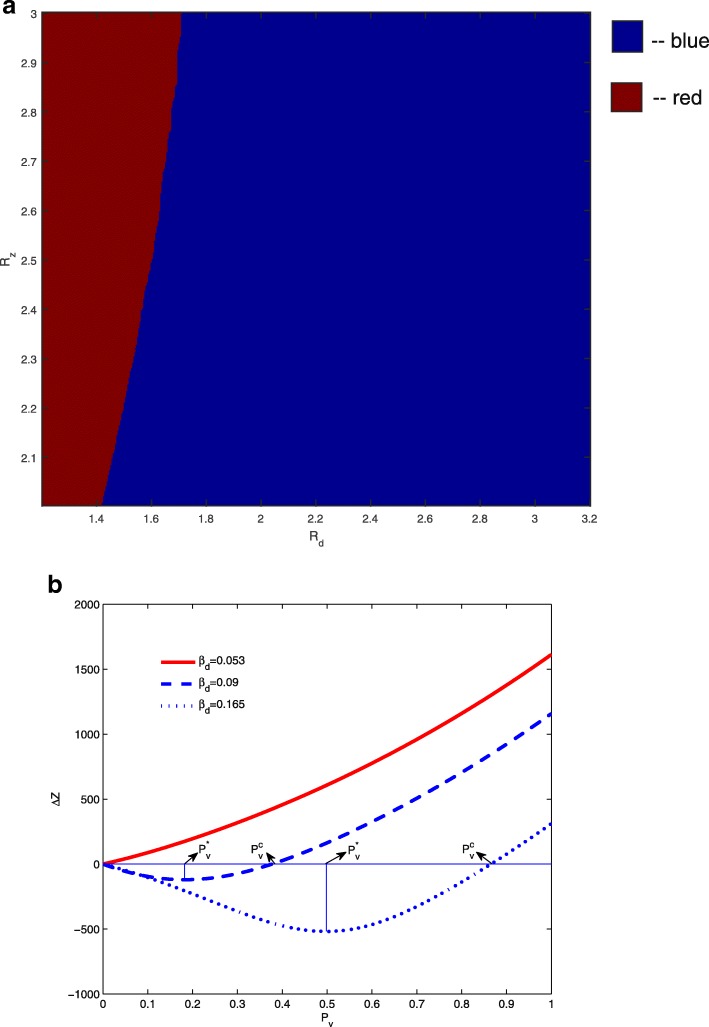
Regions of *R*_*d*_ and *R*_*z*_ where dengue vaccination increases/reduces Zika infections. **a** The red curve represents the variation of *ΔZ* with respect to *P*_*v*_ when *R*_*z*_ and *R*_*d*_ falls into the red region in (**b**). The blue curves refer to the case when *R*_*z*_ and *R*_*d*_ fall into the blue region, $P_{v}^{*}$ and $P_{v}^{c}$ are the optimal and critical dengue vaccine rates to reduce Zika infections. Here *κ*=2. **b** Dengue vaccine can always increase Zika infections if the basic reproduction numbers *R*_*d*_ and *R*_*z*_ are in the red region. The vaccine can reduce Zika infections if the vaccine coverage rate is within a certain interval when *R*_*d*_ and *R*_*z*_ are in the blue region

We denote the final size of Zika infections with and without dengue vaccination as *Z*_*a*_ and *Z*_*b*_, respectively. Thus, the difference of the accumulated numbers of Zika infections with and without dengue vaccine is *ΔZ*=*Z*_*a*_−*Z*_*b*_. We fix *β*_*z*_=0.18 and *κ*=2, and plot the variation of *ΔZ* with respect to the effective dengue vaccine coverage rate *P*_*v*_ in Fig. 2a for various *β*_*d*_ values. When *β*_*d*_=0.053, any level of dengue vaccine coverage will trigger increased Zika infections (i.e. *ΔZ*>0 for 0<*P*_*v*_≤1). For the cases of *β*_*d*_=0.09 and *β*_*d*_=0.165, we note that the plotted curve for the total number of Zika infections prevented from dengue vaccination (shown in Fig. 2a) switches only once from negative to positive at $P_{v}=P_{v}^{c}$. Therefore, $P_{v}^{c}$ is the critical dengue vaccine coverage rate above which dengue vaccination increases Zika infections in the population, and we can numerically locate $P_{v}^{*}$, the optimal dengue vaccine coverage rate for the maximal reduction of Zika infections. Thus, $\left [0,P_{v}^{c}\right ]$ can be regarded as a safe interval of effective dengue vaccine coverage for managing a Zika outbreak.

**Fig. 3:**
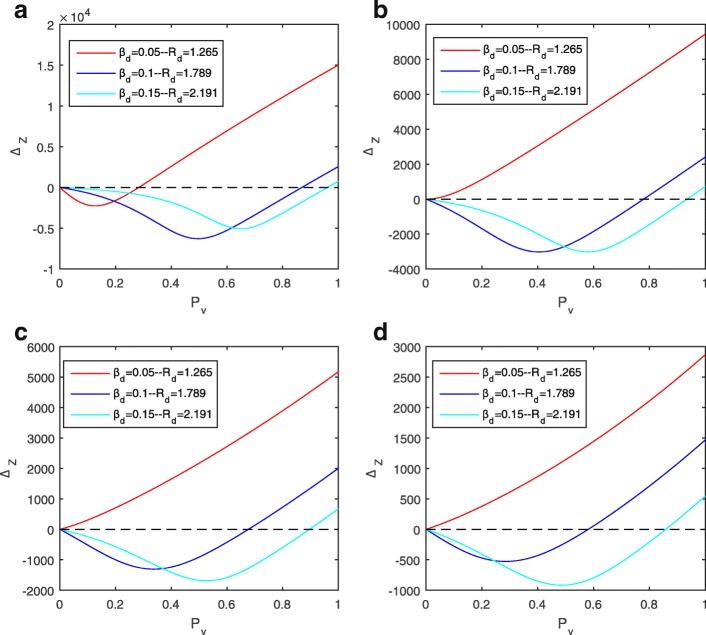
*Δ*_*Z*_ curves under various parameter sets. **a** *β*_*z*_=0.06; **b** *β*_*z*_=0.09; **c** *β*_*z*_=0.12; **d** *β*_*z*_=0.15

With fixed values of *β*_*d*_ and *β*_*z*_, we can determine the basic reproduction numbers *R*_*d*_ and *R*_*z*_. For example, *R*_*d*_=1.7 when *β*_*d*_=0.09 and *R*_*z*_=2.4 when *β*_*z*_=0.18. Therefore, for any pair of *R*_*d*_ and *R*_*z*_, we can numerically determine the shape of *ΔZ* curve with respect to *P*_*v*_ and are able to show that the curve is either non-negative (as the red curve in Fig. 2a) or switches from being negative to positive only once (as the blue curves in Fig. 2a). Numerical examples with various parameter sets are provided in Fig. 3. Consequently, we can obtain the critical and optimal dengue vaccine coverage rates for a Zika outbreak for the cases as the blue curves in Fig. 2a. These simulations show that whether a dengue vaccine, in the presence of ADE, will increase the final size of Zika infections depends on the relative ratio of the two basic reproduction numbers *R*_*d*_ and *R*_*z*_. This is shown in Fig. 2b when *κ* is fixed to be 2. In the blue area, the total number of Zika infections will be reduced when the (effective) vaccination rate *P*_*v*_ is within a range up to $P_{v}^{c}$, exceeding which dengue vaccine will be counter-productive in terms of controlling Zika infections.

**Fig. 4:**
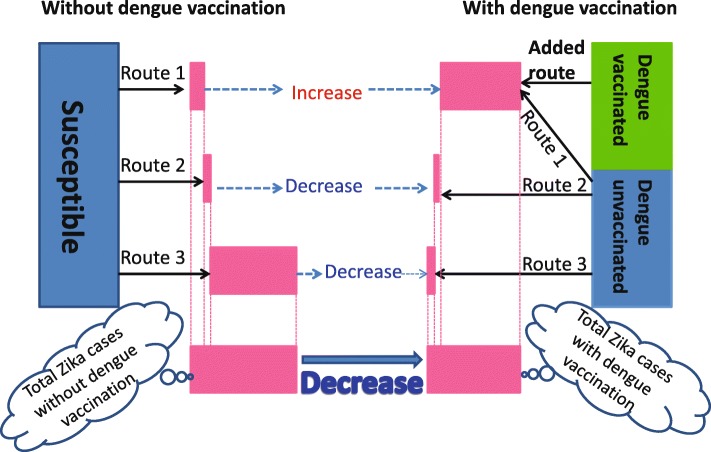
Zika transmission diagram showing how dengue vaccination affects the total number of Zika infections by the end of an outbreak. Through Route 1 individuals are directly infected by ZIKV without a previous dengue infection; through Added Route individuals are infected by ZIKV with a prior dengue vaccination; through Route 2 individuals are coinfected with Zika and dengue; and through Route 3 individuals are subsequently infected by ZIKV with a prior recovery from dengue infection. The width of the pink bars, based on model simulations, represent the contributions toward Zika infections from each route and the total Zika infections of the two scenarios. While the accumulated number of human infected with ZIKV could increase significantly through Route 1 and Added Route with enhanced transmission of ZIKV after dengue vaccine and due to ADE, because of the cocirculation of dengue and Zika viruses, the accumulated number of ZIKV infections can decrease significantly through Route 2 or/and Route 3 since the susceptible humans who can gain Zika through Route 2 and/or Route 3 are proportionally decreased
- Route 2 - coinfection by dengue and Zika (i.e. *S*→*I*_*d*_→*I*_*dz*_ and *S*→*I*_*dz*_);
- Route 3 - infection by ZIKV with prior recovered dengue infection $\left (\text {i.e. } S\to I_{d}\to R^{d}\to J_{d}^{z}\right)$.

To gain insights about why dengue vaccination can reduce the final size of ZIKV infections, we notice that there are multiple pathways towards ZIKV infection in the presence of ADE when a dengue vaccine is used in the population. This is illustrated in Fig. 4. The left panel of Fig. 4 shows the three pathways without dengue vaccine:

- Route 1 - infection by ZIKV without prior dengue infection (i.e. *S*→*I*_*z*_);

The right panel shows four pathways with dengue vaccine, with one extra route:

- Added Route - infection by ZIKV with prior dengue vaccination $\left (\text {i.e. } \hat {R}^{d}\to J_{d}^{z}\right)$.

In the scenario with dengue vaccination, we will regard Route 1A as the combination of Zika cases through both Route 1 and Added Route. Thus we can compare cases through Route 1A under dengue vaccination scenario with the cases through Route 1 under the scenario without vaccination.

**Fig. 5:**
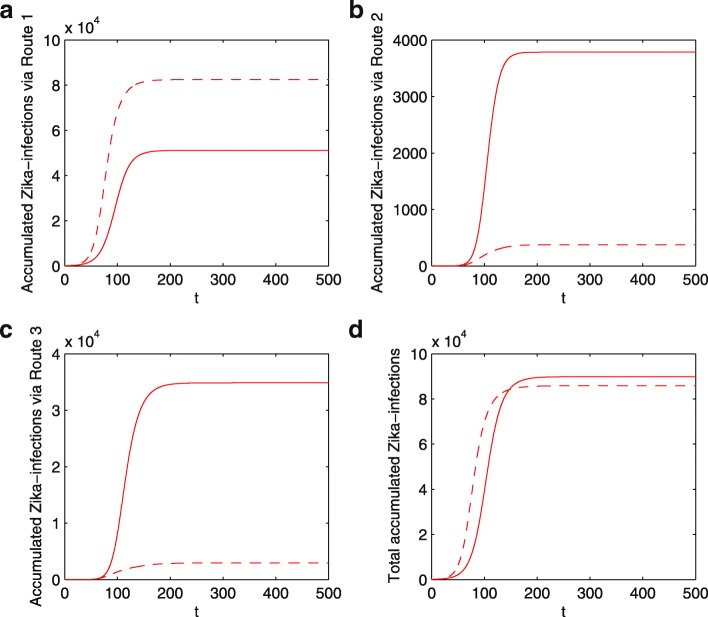
Accumulated Zika infections via each transmission route. **a** through Route 1 (individuals are infected by ZIKV with no prior dengue infection) for the case without vaccination; through Route 1A (individuals are infected by ZIKV either with no prior dengue infection or with prior dengue vaccination) for the case with vaccination; **b** through Route 2 (individuals are coinfected with Zika and dengue); **c** through Route 3 (individuals, who were infected with dengue previously and recovered, are subsequently infected with ZIKV); **d** through all routes. The dash curves denote the number with dengue vaccine while the solid curves are the number without dengue vaccine. Here we fix *β*_*d*_=0.07, *β*_*z*_=0.07, *κ*=2 and *P*_*v*_=0.3

**Fig. 6:**
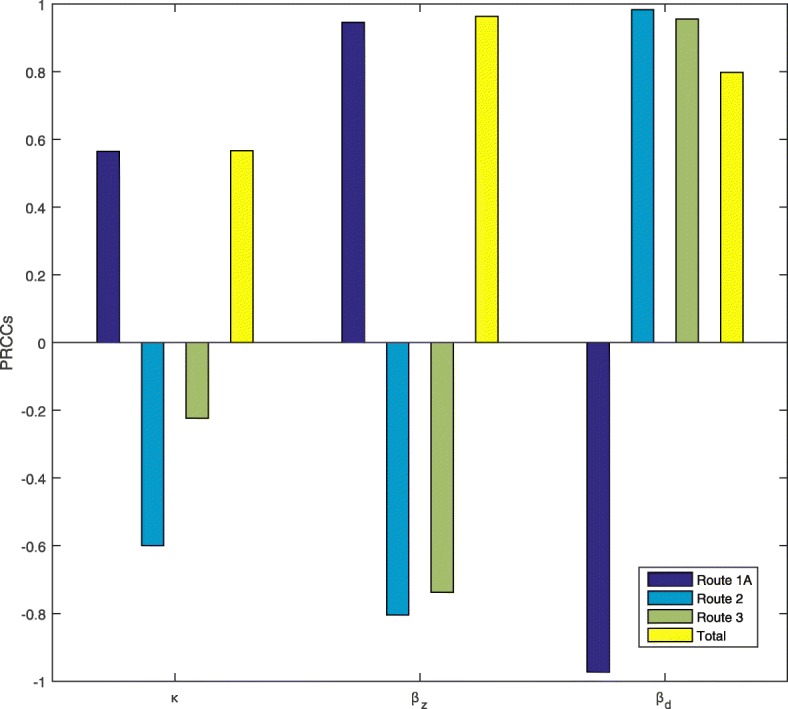
Sensitivity analysis on Zika infections via all transmission routes. PRCCs of the cumulative Zika infections through Route 1, Route 2, Route 3, and the total number of cases, with parameters uniformly distributed in the ranges *κ*∈[1,3], *β*_*z*_∈[0.125,0.281] and *β*_*d*_∈[0.045,0.32]. *P*_*v*_=50*%* is fixed in this analysis

A dengue vaccination program, while likely reducing the dengue control reproduction number $R_{d}^{P_{v}}$, always increases the Zika control reproduction number $R_{z}^{P_{v}}$. This is intuitively true and is clearly shown in the analytic formula. Therefore, the initial Zika growth rate is always increased due to ADE and this increases the number of Zika infections initially. Counter-intuitively, the total (accumulated) number of Zika infections by the end of an outbreak can be reduced after the dengue vaccine and due to ADE as shown in Fig. 5d. With an increase in *R*_*z*_ after dengue vaccine and due to ADE, the accumulated number of humans infected with ZIKV could increase significantly through Route 1A compared with the case number through Route 1 without dengue vaccine, as shown in Fig. 5a. However, the accumulated number of Zika infections can also decrease significantly through Route 2 and/or Route 3 due to the proportional decrease of the susceptible populations who can get Zika infection through Route 2 and/or Route 3, as shown in Fig. 5b,c. Therefore, it is not hard to understand the area separation in Fig. 2b: dengue vaccination always increases ZIKV infections through Route 1A but decreases ZIKV infections through Route 2 or/and Route 3, with any fixed *R*_*z*_, when *R*_*d*_ is larger, ZIKV infections through Route 2 or/and Route 3 will decrease significantly, resulting a decrease in total ZIKV infections. We also perform sensitivity analysis of the Zika case numbers through Route 1A, Route 2, Route 3, and the total with respect to *R*_*d*_,*R*_*z*_, *κ* in Fig. 6. It can be seen that the number of Zika infections through all three routes are very sensitive to these three parameters.

### Optimizing dengue vaccination to reduce Zika infections

**Fig. 7:**
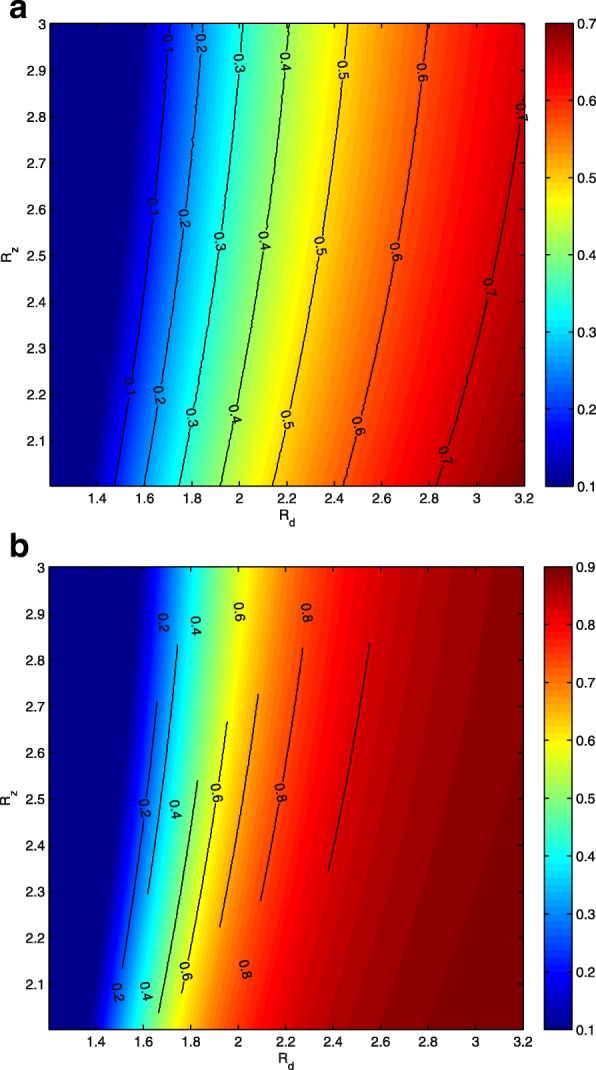
Contour plots of the critical and optimal dengue vaccine coverage rates with respect to *R*_*d*_ and *R*_*z*_. **a** Contour plot of the optimal dengue vaccine coverage rate. **b** Contour plot of the critical dengue vaccine coverage rate. Here *κ*=2

**Fig. 8:**
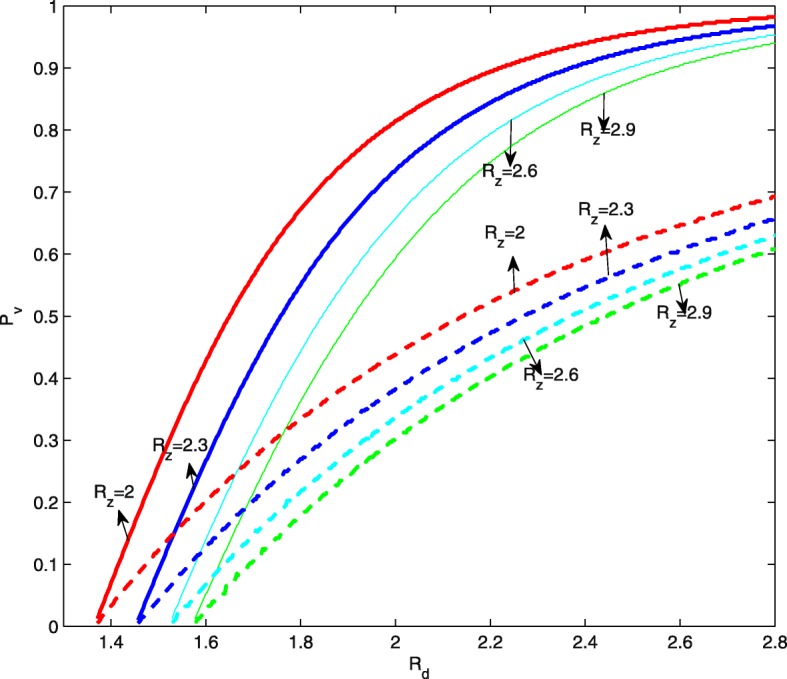
Critical and optimal dengue vaccine coverage rates under four special cases. The solid and dash curves represent the changing relationship of the critical and optimal values of dengue vaccine coverage rate with respect to the basic reproduction number for dengue *R*_*d*_, respectively. Here the antibody-dependent enhancement rate is *κ*=2

With the same ranges of *R*_*d*_ and *R*_*z*_ in Fig. 2b, Fig. 7b shows the counter plot of the critical effective dengue vaccine coverage rate versus *R*_*d*_ and *R*_*z*_. It indicates that the critical value increases as *R*_*d*_ increases or *R*_*z*_ decreases. Similar results can be obtained for the optimal dengue vaccine coverage rate, as shown in Fig. 7a. It can be seen more clearly from Fig. 8 that if we fix *R*_*z*_, then the safe interval of the dengue vaccine coverage rate can be enlarged as *R*_*d*_ increases. Correspondingly, the optimal dengue vaccine coverage rate can also be increased to minimize the accumulated number of Zika infections. For example, if we fix *R*_*z*_=2, then the critical effective dengue vaccine coverage rate increases from 0 to 98% while the optimal dengue vaccine coverage rate increases from 0 to 70%.

**Fig. 9:**
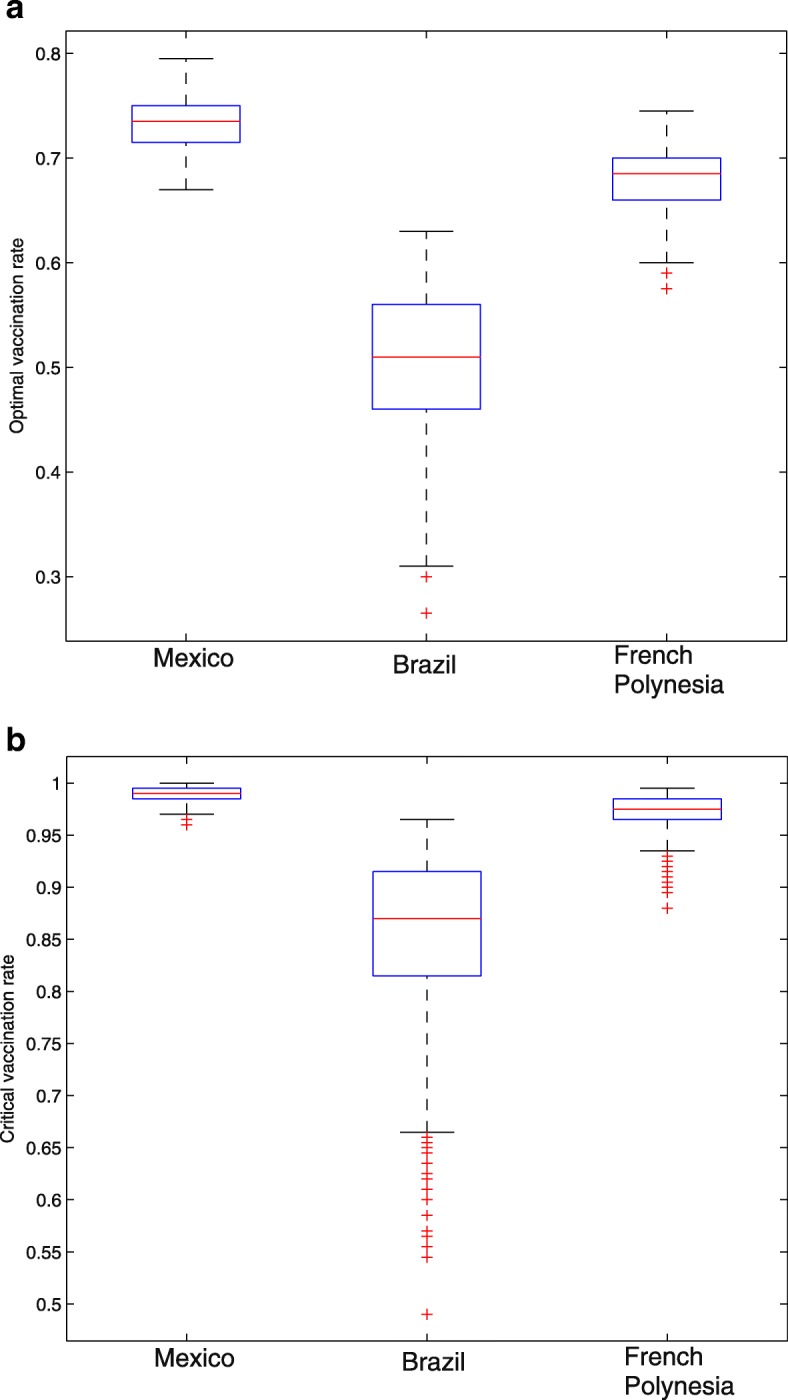
Sensitivity analysis on optimal and critical vaccine coverages. **a** optimal vaccine coverage rate; **b** critical vaccine coverage rate. For each country shown in the figure, we sample the ADE factor *κ* in a large range of [1.2,2.5], and the corresponding local Zika and dengue basic reproduction numbers estimated from literatures. Each box plot has its first, second, and third quartiles marked. We sample (*R*_*d*_,*R*_*z*_) from ranges cited from literatures: [2.89,3.29]×[2,2.3] ; [2.07,2.6]×[2.13,2.53]; and [2.73,3.13]×[2.13,2.53]

Past studies have estimated the Zika basic reproduction number to be 2.33 (95% CI 2.15-2.51) in French Polynesia [39], 2.33 (95% CI 1.97-2.97) in Rio de Janeiro, Brazil [40], and 2.1 in Mexico [41]. The dengue basic reproduction number has also been estimated to be 2.93 (95% CI 1.89-5.47) in French Polynesia [42], 2.32 (95% 2.07-2.60) in Brazil [42], and 3.09 (95% CI 2.34-3.84) in Mexico [43]. With these estimated basic reproduction numbers in pair (basic reproduction number for Zika, and basic reproduction number in dengue that is certainly serotype-specific), we are able to estimate the optimal and critical effective dengue vaccine coverage rates for relevant regions with similar parameter ranges ranges. Figure 9 shows that our estimation of the optimal and critical effective vaccine coverage is robust with estimation or measurement errors of *κ*∈[1.2,2.5] and (*R*_*d*_,*R*_*z*_) varying in small intervals around the above literature estimated values for Mexico, Brazil, and French Polynesia. We would like to point out that, due to the lack of information, we pick the values of (*R*_*d*_,*R*_*z*_) for the three regions from different literatures where different estimation methodologies were applied. Our estimation for vaccination coverages depends highly on the quality of the estimated values of (*R*_*d*_,*R*_*z*_), hence on the epidemiological study of the involved diseases or serotypes.

### The case of antibody-dependent neutralization (ADN)

**Fig. 10:**
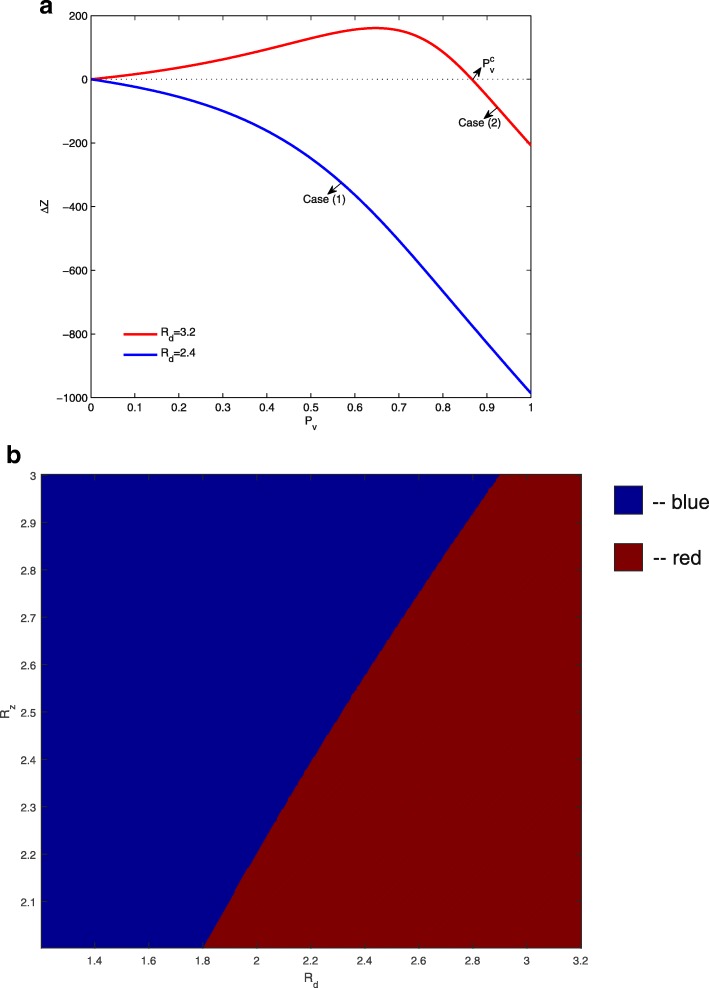
Scenarios for antibody-dependent neutralization with *κ*=0.7. Here the Zika basic reproduction number is fixed as 2.7. **a** Variation of *ΔZ* with respect to dengue vaccine coverage rate *P*_*v*_. **b** Regions of *R*_*d*_ and *R*_*z*_ where dengue vaccination affects Zika infections in two ways as illustrated in (**a**)

Our model also allows us to examine the case where *κ*<1, the convalescent plasma following dengue infection can partially protect humans from being infected with Zika. We find that there may be two typical cases for the curve of *ΔZ* with respect to *P*_*v*_ as shown in Fig. 10 where *κ*=0.7. Case (1): Dengue vaccine can always reduce the accumulated number of Zika infections; Case (2): Dengue vaccine increases the accumulated number of Zika infections when the dengue vaccine coverage rate is below a critical value (denoted by $P_{v}^{c}$), while it can decrease the accumulated number of Zika infections when the dengue vaccine coverage rate exceeds the critical value. In Fig. 10b, we plot the different regions corresponding to the two cases with the basic reproduction numbers varying in the same way as in Fig. 2b. Note that for case (1) vaccination against dengue is always beneficial to Zika, but for Case (2) the safe interval of the dengue vaccine coverage rate should be $\left [P_{v}^{c},1\right ]$.

## Discussion

There is increasing evidence of immunological cross-reactivity between dengue and Zika viruses, which indicates that convalescent plasma following dengue infection can enhance the ZIKV infection. Through a conceptual mathematical model, we addressed the issue that vaccination against dengue may increase Zika infections in the presence of ADE. We found that under some conditions, dengue vaccination can reduce Zika infections. We computed explicitly the parameter ranges within which dengue vaccine increases or reduces Zika infections and examined how these results depend on the basic reproduction numbers of both diseases.

In Fig. 2a, where three different values of *β*_*d*_ were chosen while other parameters were fixed, we found two different types of impact of dengue vaccination: it either always increases Zika infections, or can reduce Zika infections when the vaccine coverage rate is in a safe interval. We illustrated in Fig. 2b that our models enable us to determine which scenario can occur when *R*_*d*_ varies between 1.2 and 3.2 while *R*_*z*_ varies from 2 to 3.

The optimal and critical effective dengue vaccine coverage rates can be calculated through numerical simulations for regions where both *R*_*d*_ and *R*_*z*_ can be estimated. Figure 7 gives the dengue vaccine isoclines, panel (a) for optimal and panel (b) for critical rates. This clearly shows that in the regions with similar dengue epidemics (same *R*_*d*_), the higher the *R*_*z*_ the smaller the optimal dengue vaccine coverage rate; and in the regions with similar Zika epidemics (same *R*_*z*_), the higher the *R*_*d*_ the higher the optimal dengue vaccine coverage rate. For example, in the area with the pair of basic reproduction numbers (*R*_*d*_,*R*_*z*_) near (3.09, 2.1), (2.32, 2.33) and (2.93, 2.33), the optimal and critical effective dengue vaccine coverage rates are (73.6%, 99%), (51.4%, 88.2%), and (68.2%, 97.6%), respectively. If we use the efficacy of the dengue vaccine 71.6 and 76.9% for serotypes 3 and 4, 54.7 and 43.0% for serotypes 1 and 2 previously reported [44], then the optimal effective vaccine coverage for the regions with the aforementioned basic reproduction number pairs (for Dengue and for Zika) can be achieved. Our analysis shows that if the dengue vaccine efficacy is less than 90%, high dengue vaccination coverage in these regions contribute to the control of Zika. This result, in the aspect of potential dengue vaccination impacts on Zika outbreaks, reconciles the WHO’s former position on the use of the vaccine “for highly endemic areas” [45]. We are aware that WHO has revised its position for a given vaccine product given the updated data from the tetravalent dengue vaccine producer, but we are also aware there are other potential competitive vaccine products and our model analysis can be reproduced once the efficacy of these vaccine products becomes available.

The sensitivity analysis, illustrated in Fig. 9, shows that the optimal and critical dengue vaccine coverage rates are robust to uncertainty and estimation errors of dengue and Zika epidemic characteristics, and to the assumed ADE level (*κ*). This study thus shows the promise of an integrative dengue-Zika control strategy in dengue epidemic regions with access to dengue vaccine and immunization. In the presence of antibody-dependent enhancement, caution has to be exercised to optimally design the dengue vaccine program with an appropriate coverage that can reduce the final size of Zika infections. Our study shows this optimal program is feasible.

We also investigated the impact of dengue vaccine on Zika infections if the convalescent plasma following dengue infection partially protects human from being infected by ZIKV (when *κ*<1). We concluded in Fig. 10 that the variation of *ΔZ* with respect to *P*_*v*_ becomes different than the case of *κ*>1. We found that in the case of *κ*=0.7, dengue immunization can either reduce total Zika cases regardless of vaccination coverage, or boost Zika cases with small coverages but reduce Zika cases with large coverages. Dengue vaccination becomes beneficial at any coverage level when *R*_*z*_ is larger than *R*_*d*_ to some extent.

## Limitations

It has been shown that naturally-acquired dengue infection against a single serotype can be incomplete, resulting in individuals being infected multiple times by the same serotype [46, 47]. To incorporate this incomplete and/or waning natural protection, we will need to modify our model setup to allow recovered individuals from dengue infection to become partially susceptible to dengue infection, in addition to enhanced susceptibility to Zika infection. Should new evidence arise to indicate the difference of dengue immunity from natural infection and vaccination, our model parameters need to be modified by incorporating two different *κ*. The qualitative conclusion should remain since our sensitivity analysis indicates the robustness of our conclusion with respect to the change of *κ*, but accurate optimal and critical vaccine rates may be slightly changed.

Our model captures some important aspects of dengue and Zika transmission to address the impact of dengue vaccine usage on Zika infections in a homogeneous population within a single season and in a setting only one dengue serotype is involved. This conceptual model provides a basis for future studies to incorporate other important epidemiological characteristics such as different serotypes of dengue, asymptomatic infection, generation time of secondary Zika/Dengue infections, sexually transmission of ZIKV, and variation in transmission potential and severity (and hence risk, and cost-benefit) for different age/gender groups [48]. Seasonal factors can and should also be incorporated to allow temporal variation of transmission parameters to address more logistic vaccination program design that must consider risk differentiation by gender, age and other demographic characteristics [10]. To examine the long term impact of dengue vaccine on Zika transmission, we should also consider the issue whether dengue vaccine offers only short-term protection (and hence ADE), which can be modelled by allowing recovery to the dengue-susceptible populations. Finally, in view of the recent study [49] on bidirectional ADE impact between dengue and Zika, and the substantial global efforts towards Zika vaccine development, our model should be modified by further stratification of the vector and human populations and additional cost-benefit analyses to inform “long-term high prioritisation and adequate resources” [50].

## Conclusions

In this paper, we evaluate the impact of dengue vaccination on Zika infection dynamics through a conceptual mathematical coinfection dynamics model. We show that an appropriately designed and optimized dengue vaccine usage plan can not only help control the dengue spread but also, counter-intuitively, reduce Zika infections. We also identify optimal dengue vaccination coverages for controlling dengue and simultaneously reducing Zika infections, as well as the critical coverages exceeding which dengue vaccination will increase Zika infections. This study shows the promise of an integrative dengue-Zika control strategy in dengue epidemic regions with access to dengue vaccine, the mathematical model provides the first step towards well-designed regional and global vector-borne disease immunization programs.

## References

[1] Gubler DJ (1998). Dengue and dengue hemorrhagic fever. Clin Microbiol Rev.

[2] World Health Organization (WHO). WHO Dengue and Severe Dengue, Fact Sheet No. 117, Updated May 2015. http://www.who.int/en/news-room/fact-sheets/detail/dengue-and-severe-dengue.

[3] Massad E, Burattini MN, Ximenes R, Amaku M, Wilder-Smith A (2014). Dengue outlook for the World Cup in Brazil. Lancet Infect Dis.

[4] Halstead SB (1988). Pathogenesis of dengue: challenges to molecular biology. Science.

[5] Dejnirattisai W, Jumnainsong A, Onsirisakul N, Fitton P, Vasanawathana S, Limpitikul W, Puttikhunt C, Edwards C, Duangchinda T, Supasa S (2010). Cross-reacting antibodies enhance dengue virus infection in humans. Science.

[6] Ndifon W, Wingreen NS, Levin SA (2009). Differential neutralization efficiency of hemagglutinin epitopes, antibody interference, and the design of influenza vaccines. Proc Natl Acad Sci USA.

[7] Ferguson N, Anderson R, Gupta S (1999). The effect of antibody-dependent enhancement on the transmission dynamics and persistence of multiple-strain pathogens. Proc Natl Acad Sci USA.

[8] Cummings DA, Schwartz IB, Billings L, Shaw LB, Burke DS (2005). Dynamic effects of antibody-dependent enhancement on the fitness of viruses. Proc Natl Acad Sci USA.

[9] Adams B, Holmes E, Zhang C, Mammen M, Nimmannitya S, Kalayanarooj S, Boots M (2006). Cross-protective immunity can account for the alternating epidemic pattern of dengue virus serotypes circulating in Bangkok. Proc Natl Acad Sci USA.

[10] Ferguson NM, Rodríguez-Barraquer I, Dorigatti I, Mier-y-Teran-Romero L, Laydon DJ, Cummings DA (2016). Benefits and risks of the Sanofi-Pasteur dengue vaccine: Modeling optimal deployment. Science.

[11] World Health Organization (WHO). Updated Questions and Answers Related to the Dengue Vaccine Dengvaxia and Its Use. http://www.who.int/immunization/diseases/dengue/q_and_a_dengue_vaccine_dengvaxia_use/en/.

[12] Dick GWA, Kitchen SF, Haddow AJ (1952). Zika Virus. Trans R Soc Trop Med Hyg.

[13] Duffy MR, Chen T-H, Hancock WT, Powers A, Kool JL, Lanciotti RS, Pretrick M, Marfel M, Holzbauer S, Dubray C (2009). Zika virus outbreak on Yap Island, federated states of Micronesia. N Engl J Med.

[14] Musso D, Nilles EJ, Cao-Lormeau V (2014). Rapid spread of emerging Zika virus in the Pacific area. Clin Microbiol Infect.

[15] Petersen L, Jamieson D, Powers A, Honein M (2016). Zika Virus. N Engl J Med.

[16] Campos GS, Bandeira AC, Sardi SI (2015). Zika virus outbreak, Bahia, Brazil. Emerg Infect Dis.

[17] Massad E, Tan SH, Khan K, Wilder-Smith A (2016). Estimated Zika virus importations to Europe by travellers from Brazil. Glob Health Action.

[18] Bogoch II, Brady O, Kraemer M, German M, Creatore MI, Brent S, Watts AG, Hay SI, Kulkarni MA, Brownstein JS (2016). Potential for Zika virus introduction and transmission in resource-limited countries in Africa and the Asia-Pacific region: a modelling study. Lancet Infect Dis.

[19] Bogoch II, Brady O, Kraemer M, German M, Creatore MI, Kulkarni MA, Brownstein JS, Mekaru SR, Hay SI, Groot E (2016). Anticipating the international spread of Zika virus from Brazil. Lancet.

[20] Pan American Health Organization (PAHO), World Health Organization (WHO). Zika - Epidemiological Update, 29 December 2016. https://www.paho.org/hq/dmdocuments/2016/2016-dec-29-phe-epi-update-zika-virus.pdf.

[21] Gao D, Lou Y, He D, Porco TC, Kuang Y, Chowell G, Ruan S (2016). Prevention and control of Zika as a mosquito-borne and sexually transmitted disease: a mathematical modeling analysis. Sci Rep.

[22] Besnard M, Lastere S, Teissier A, Cao-Lormeau V, Musso D (2014). Evidence of perinatal transmission of Zika virus, French Polynesia, December 2013 and February 2014. Euro Surveill.

[23] Dupont-Rouzeyrol M, O’Connor O, Calvez E, Daures M, John M, Grangeon J-P, Gourinat A-C (2015). Co-infection with Zika and dengue viruses in 2 patients, New Caledonia, 2014. Emerg Infect Dis.

[24] Pessôa R., Patriota JV, de Souza MdL, Felix AC, Mamede N, Sanabani SS (2016). Investigation into an outbreak of dengue-like illness in pernambuco, brazil, revealed a cocirculation of zika, chikungunya, and dengue virus type 1. Medicine.

[25] Dejnirattisai W, Supasa P, Wongwiwat W, Rouvinski A, Barba-Spaeth G, Duangchinda T, Sakuntabhai A, Cao-Lormeau V-M, Malasit P, Rey FA (2016). Dengue virus sero-cross-reactivity drives antibody-dependent enhancement of infection with zika virus. Nat Immunol.

[26] Priyamvada L, Quicke KM, Hudson WH, Onlamoon N, Sewatanon J, Edupuganti S, Pattanapanyasat K, Chokephaibulkit K, Mulligan MJ, Wilson PC (2016). Human antibody responses after dengue virus infection are highly cross-reactive to Zika virus. Proc Natl Acad Sci USA.

[27] Bardina SV, Bunduc P, Tripathi S, Duehr J, Frere JJ, Brown JA, Nachbagauer R, Foster GA, Krysztof D, Tortorella D (2017). Enhancement of Zika virus pathogenesis by preexisting antiflavivirus immunity. Science.

[28] Pierson TC, Graham BS (2016). Zika virus: immunity and vaccine development. Cell.

[29] Valentine G, Marquez L, Pammi M (2016). Zika virus epidemic: an update. Expert Rev Anti-Infect Ther.

[30] Tang B, Xiao Y, Wu J (2016). Implication of vaccination against dengue for Zika outbreak. Sci Rep.

[31] Van den Driessche P, Watmough J (2002). Reproduction numbers and sub-threshold endemic equilibria for compartmental models of disease transmission. Math Biosci.

[32] Wearing HJ, Rohani P (2006). Ecological and immunological determinants of dengue epidemics. Proc Natl Acad Sci USA.

[33] Recker M, Blyuss KB, Simmons CP, Hien TT, Wills B, Farrar J, Gupta S (2009). Immunological serotype interactions and their effect on the epidemiological pattern of dengue. Proc R Soc Lond B Biol Sci.

[34] Charles AS, Christofferson RC. Utility of a dengue-derived monoclonal antibody to enhance Zika infection in vitro. Edition 1. PLoS Curr Outbreaks. 2016. https://doi.org/10.1371/currents.outbreaks.4ab8bc87c945eb41cd8a49e127082620https: //doi.org/10.1371/currents.outbreaks.4ab8bc87c945eb41cd8a49e127082620. 10.1371/currents.outbreaks.4ab8bc87c945eb41cd8a49e127082620 PMC5026288 27660733

[35] Andraud M, Hens N, Marais C, Beutels P (2012). Dynamic epidemiological models for dengue transmission: a systematic review of structural approaches. PLoS ONE.

[36] Towers S, Brauer F, Castillo-Chavez C, Falconar AK, Mubayi A, Romero-Vivas CM (2016). Estimate of the reproduction number of the 2015 Zika virus outbreak in Barranquilla, Colombia, and estimation of the relative role of sexual transmission. Epidemics.

[37] Kucharski AJ, Funk S, Eggo RM, Mallet H-P, Edmunds WJ, Nilles EJ (2016). Transmission dynamics of Zika virus in island populations: a modelling analysis of the 2013–14 French Polynesia outbreak. PLoS Negl Trop Dis.

[38] Majumder MS, Cohn E, Fish D, Brownstein JS (2016). Estimating a feasible serial interval range for Zika fever. Bull World Health Organ.

[39] Zhang Q, Sun K, Chinazzi M, Pastore-Piontti A, Dean NE, Rojas DP, Merler S, Mistry D, Poletti P, Rossi L, Bray M, Halloran ME, Longini IM, Vespignani A. Projected spread of Zika virus in the Americas. bioRxiv. 2016. https://doi.org/10.1101/066456.

[40] Villela DAM, Bastos L, Carvalho LM, Cruz OG, Gomes MFC, Durovni B, Lemos MC, Saraceni V, Coelho FC, Codeco CT (2017). Zika in Rio de Janeiro: Assessment of basic reproduction number and comparison with dengue outbreaks. Epidemiol Infect.

[41] Rocklöv J, Quam MB, Sudre B, German M, Kraemer M, Brady O, Bogoch II, Liu-Helmersson J, Wilder-Smith A, Semenza JC (2016). Assessing seasonal risks for the introduction and mosquito-borne spread of Zika virus in Europe. EBioMedicine.

[42] Imai N, Dorigatti I, Cauchemez S, Ferguson NM (2015). Estimating dengue transmission intensity from sero-prevalence surveys in multiple countries. PLoS Negl Trop Dis.

[43] Chowell G, Diaz-Duenas P, Miller J, Alcazar-Velazco A, Hyman J, Fenimore P, Castillo-Chavez C (2007). Estimation of the reproduction number of dengue fever from spatial epidemic data. Math Biosci.

[44] World Health Organization (WHO). Questions and Answers on Dengue Vaccines. http://www.who.int/immunization/research/development/dengue_q_and_a/en/.

[45] World Health Organization (WHO). Updated Questions and Answers Related to Information Presented in the Sanofi Pasteur - Press Release on 30 November 2017 with Regards to the Dengue Vaccine Dengvaxia. http://www.who.int/immunization/QA-dengue-vaccine.pdf.

[46] Forshey BM, Reiner RC, Olkowski S, Morrison AC, Espinoza A, Long KC, Vilcarromero S, Casanova W, Wearing HJ, Halsey ES (2016). Incomplete protection against dengue virus type 2 re-infection in Peru. PLoS Negl Trop Dis.

[47] Waggoner JJ, Balmaseda A, Gresh L, Sahoo MK, Montoya M, Wang C, Abeynayake J, Kuan G, Pinsky BA, Harris E (2016). Homotypic dengue virus reinfections in nicaraguan children. J Infect Dis.

[48] Flasche S, Jit M, Rodríguez-Barraquer I, Coudeville L, Recker M, Koelle K, Milne G, Hladish TJ, Perkins TA, Cummings DA (2016). The long-term safety, public health impact, and cost-effectiveness of routine vaccination with a recombinant, live-attenuated dengue vaccine (dengvaxia): a model comparison study. PLoS Med.

[49] Kawiecki AB, Christofferson RC (2016). Zika Virus–Induced Antibody Response Enhances Dengue Virus Serotype 2 Replication In Vitro. J Infect Dis.

[50] Pang T, Mak TK, Gubler DJ (2017). Prevention and control of dengue—the light at the end of the tunnel. Lancet Infect Dis.

